# Anti-dipeptidyl-peptidase-like protein-6 encephalitis with late-onset gastric cancer: A case report and 2-year follow-up

**DOI:** 10.1097/MD.0000000000041534

**Published:** 2025-02-14

**Authors:** Guolu Wu, Yanhui Shi, Jialan Sun, Feifei Wu, Mei Jiang, Qiang Li

**Affiliations:** a Department of Neurology, Shanghai Gongli Hospital of Pudong New Area, Shanghai, China; b Department of Neurology, Xuhui District Central Hospital, Shanghai, China.

**Keywords:** anti-dipeptidyl-peptidase-like protein-6 encephalitis, Epstein-Barr virus infection, gastric cancer, psychiatric symptoms

## Abstract

**Rationale:**

Anti-dipeptidyl-peptidase-like-protein 6 (DPPX) encephalitis is a rare form of autoimmune encephalitis, with no more than 80 cases reported to date. Cases of anti-DPPX encephalitis comorbid malignancy are exceedingly rare. Limited cases and diverse clinical presentations bring difficulties in the understanding of this disease, including the etiology, diagnosis, treatment, and prognosis. Herein, we report a case of insidious-onset anti-DPPX encephalitis in a patient with a prior history of Epstein-Barr Virus (EBV) meningitis diagnosed with gastric cancer after 2 years of follow-up.

**Patient concerns:**

A 72-year-old emaciated male presented with recurrent seizures over the last 20 years, personality changes over 10 years, and memory loss lasting 2 years.

**Diagnoses:**

The patient was diagnosed with epilepsy, psychiatric symptoms (agitation and irritability), and mild cognitive impairment. Anti-DPPX encephalitis and prior EBV infection were ultimately diagnosed based on the combination of his symptoms and positive DPPX antibody in serum (titer 1:10) and positive EBV-IgG antibody in cerebrospinal fluid.

**Interventions:**

The patient received a course of intravenous methylprednisolone and oral sodium valproate to treat the seizures.

**Outcomes:**

After 10 days of treatment, no seizures reoccurred, although the psychiatric symptoms persisted, and his serum antibody against DPPX was still positive (titer 1:10). Unfortunately, the family members asked for the patient to be discharged automatically and refused oral steroids after discharge. Through regular telephone follow-ups for 2 years after discharge, we learned that the patient did not experience any similar convulsions but still showed irritability when administered perphenazine. Unfortunately, the patient had gastric cancer with multiple metastases and was receiving palliative care.

**Lessons:**

This report illustrates a rare case of EBV meningitis in childhood, resulting in a long-standing, stable course of anti-DPPX encephalitis, and subsequent gastric cancer. This case broadens the atypical presentation spectrum of anti-DPPX encephalitis and emphasizes the need for screening for malignant tumors, including lymphoma, nasopharyngeal carcinoma, and gastric cancer, particularly in patients with latent EBV infection.

## 1. Introduction

Dipeptidyl-peptidase-like protein-6 (DPPX) antibody-associated encephalitis is a rare form of autoimmune encephalitis (AIE) associated with the presence of antibodies against DPPX, which can cause a series of symptoms, including cognitive dysfunction, neuropsychiatric symptoms, and central nervous system (CNS) hyperexcitability, commonly preceded by prodromal weight loss and diarrhea. However, some patients do not have clear gastrointestinal symptoms, while the lack of typical imaging and neuroelectrophysiological representations make diagnosis difficult in the early phase of the disease. Moreover, it remains unclear whether anti-DPPX encephalitis is related to postinfectious autoimmune processes or is similar to a paraneoplastic disorder, based on limited cases. Herein, we report a case of insidious-onset anti-DPPX encephalitis with a history of Epstein-Barr virus (EBV) meningitis, diagnosed as gastric cancer after 2 years of follow-up.

## 2. Case presentation

The patient was a 72-year-old emaciated man. He was admitted to the hospital on August 9, 2021, because of recurrent tonic-clonic seizures for 20 years and confusion lasting several hours. In the past 2 decades, the patient has suffered from recurrent tonic-clonic seizures without any obvious cause, presenting as left-limb twitching, accompanied by a tilted head and gazed eyes, loss of consciousness, and incontinence. Each episode lasted for 3 to 5 minutes, with a frequency of 2 to 3 seizures per year. When seizures occurred, he was sent to a hospital and received treatment, but refused long-term medication after waking up. In the past 10 years, family members had felt that the patient’s personality has changed significantly, predominantly manifesting as agitation and irritability. The patient had experienced memory loss over the past 2 years. Several hours before hospitalization, the patient was found to be unresponsive and had slipped off a chair without limb convulsions. Regarding his medical history, his family members reported that the patient was usually thin and had loose stools 2 to 3 times a day, denying severe diarrhea. Furthermore, the patient had a history of meningitis in his 10s, and after treatment, he improved without sequelae. The patient had no history of hypertension, diabetes, head injury, or drug allergies. His developmental and family history was normal.

Upon admission, the patient’s body mass index was 17.6 kg/m^2^. Neurological examination revealed a shallow coma, a Glasgow coma scale score of 7/15, normal-sized pupils with sensitive light reflection, and bilateral Babinski sign (−). The patient’s consciousness gradually cleared approximately 39 hours following oxygen inhalation and intravenous infusion of medications. Physical reexamination revealed a Glasgow coma scale score of 15/15 (slightly excited and irritable) and a Mini-Mental State Examination score of 27/30.

**Figure 1.**
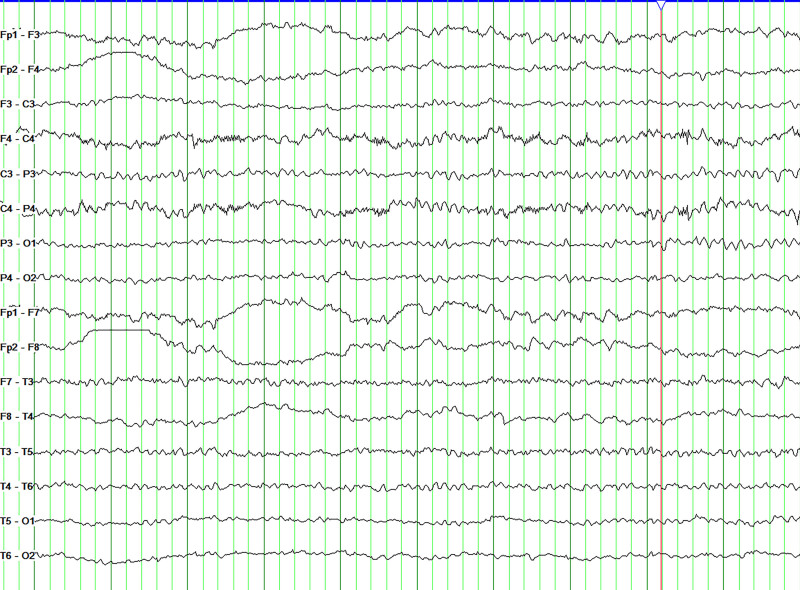
No epileptiform discharges were found in 24-h video electroencephalogram (EEG) in this patient. We arranged 2 dynamic EEG tests within 3 days to detect EEG waves, but none of them detected any epileptic waves. Figure 1 shows one of the fragments.

**Figure 2.**
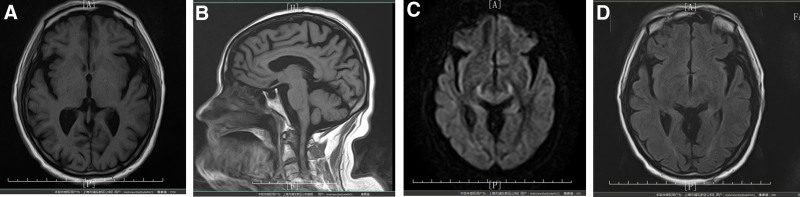
Cranial magnetic resonance imaging (MRI) showed mild atrophy in temporal lobe, cerebellar, and limbic area. T1 sequence (A and B), DWI sequence (C), and T2 FLAIR sequence (D).

**Figure 3.**
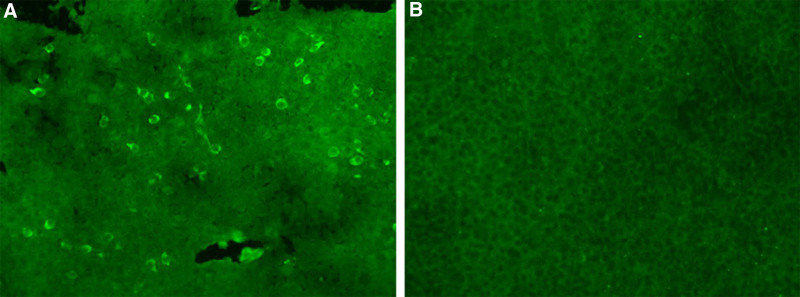
Positive reaction with transfected HEK293 cells expressing DPPX after incubation with the patient’s serum (A) (titer 1:10, CBA) and anti-DPPX negative control (B). CBA = cell-based assay, DPPX = dipeptidyl-peptidase-like protein-6.

During hospitalization, no epileptiform discharges were identified on a 24-hour video electroencephalogram (Fig. 1). Cranial magnetic resonance imaging revealed mild limbic system atrophy (Fig. 2). Routine urinalysis, blood, and stool tests showed no abnormal findings. All tumor markers were within the normal ranges. Lumbar puncture revealed colorless, transparent, and clear cerebrospinal fluid (CSF), with a pressure of 110 mmH_2_O. Cell counts and protein and glucose levels were all within the normal range. A series of antibodies and viruses were screened using a cell-based assay, which revealed that the antibody against DPPX was present (titer of 1:10) in the serum (Fig. 3). EBV virus immunoglobulin G (IgG) antibody was detected in the CSF. The results showed the same oligoclonal bands bands in both the CSF and serum. Based on the patient’s clinical features, physical findings, lab tests, and MR findings, a final diagnosis of anti-DPPX encephalitis and comorbid prior EBV infection was made.

## 3. Therapeutic intervention and outcome

**Figure 4.**
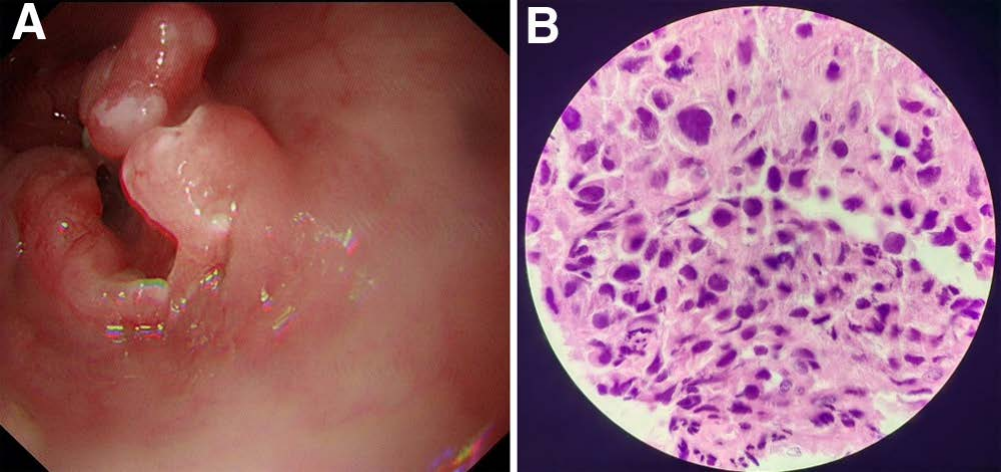
Gastroscopy pathology suggests lower esophageal adenocarcinoma (A and B).

The patient received an intravenous course of methylprednisolone (500 mg/day for 5 days, 240 mg/day for 3 days, and 120 mg for 1 day). During hospitalization, he experienced 2 tonic-clonic seizure episodes, for which sodium valproate (400 mg tid po) was administered. Following 10 days of treatment, the patient still exhibited psychiatric symptoms; however, no seizures reoccurred. Anti-DPPX antibody levels were reexamined, showing a positive result (titer 1:10, cell-based assay). Unfortunately, the family members asked for the patient to be discharged automatically and refused oral steroids after discharge. One month later, during telephone follow-up, the patient did not experience any similar seizures. After 1 year of follow-up, the patient did not have epilepsy recurrence, still showed irritability, and was administered perphenazine, but refused to undergo further major reexamination. Two years after discharge, telephone follow-up revealed that the patient had a gastric malignancy (Fig. 4) with multiple metastases, diagnosed in May 2023, and was being treated with palliative care.

## 4. Discussion

Anti-DPPX encephalitis is a rare AIE characterized by the formation of autoantibodies against DPPX, a cell surface subunit of Kv4.2 channels in the CNS expressed in the cerebellum, hippocampus, and myenteric plexus. DPPX is recognized to mediate a transient K+ current so that it plays a direct role in the firing of action potentials.^[1]^

To date, no more than 80 cases of anti-DPPX encephalitis have been reported.^[2–6]^ The classic triad of clinical symptoms includes diarrhea, weight loss, cognitive dysfunction, and CNS hyperexcitability. The progression of symptoms ranges from subacute to more insidious, occurring at a median of 8 months in one series.^[7]^ A prior literature review showed that over half of all identified patients had all 3 common classical symptoms.^[2]^ However, as an increasing number of cases have been reported, anti-DPPX encephalitis is being recognized as a disease with a broad phenotype and multifocal neurological involvement, with symptoms including central hyperexcitability (hyperekplexia and seizures), psychiatric symptoms (agitation, paranoia, hallucinations, anxiety, mutism, and depression), movement disorders (ataxia, tremor, myoclonus, rigidity, stiffness, and dystonia), dysautonomia manifestations, brainstem disorders (eye movement disturbances, dysphagia, dysarthria, and respiratory failure), dysautonomia manifestations (constipation, thermoregulation, urine retention, and tachycardia), and sleep disorder.^[5,8]^ Most patients appear to have normal or nonspecific magnetic resonance imaging findings. Similar to other types of AIC, the diagnosis of anti-DPPX encephalitis relies on a positive DPPX antibody in the serum or CSF.^[9]^ This case report describes the diagnosis of anti-DPPX encephalitis in an emaciated patient with seizures, prominent psychiatric symptoms (agitation and irritability), and mild cognitive impairment, characterized by insidious onset and a long-standing stable course of over 20 years, which has seldom been reported.

In addition to the atypical clinical features, the patient in this case notably had prior EBV meningitis deduced from positive EBV IgG in the CSF, and a delicate medical history of viral meningitis. According to epidemiological research, EBV infection is prevalent among children in China, with an incidence of over 20%.^[10]^ Following primary infection, EBV may host B lymphocytes and epithelial cells, resulting in a long-term or even lifelong state of latent infection.

Prior studies have shown that EBV can replicate in the CNS and disrupt the blood–brain barrier (BBB).^[11]^ Infected B cells are believed to have the ability to cross the BBB and the potential to differentiate into certain antibody-secreting plasma cells, which may damage neurons or glial cells. Latent EBV infection in the CNS may further induce neuroinflammation and demyelination, thereby contributing to the occurrence and development of neurological diseases, such as multiple sclerosis, acute disseminated encephalomyelitis, and AIE. Furthermore, prodromal EBV may reactivate, and the subsequent cascade may trigger and boost the immune response or facilitate autoantibodies in certain pathological backgrounds. Some AIC cases, such as N-methyl-D-aspartate-associated encephalitis, can also coincide with EBV.^[12]^ However, we were unable to make the same inference regarding the underlying mechanism of EBV-related immunopathogenesis in this case of anti-DPPX encephalitis due to the lack of positive EBV IgM in the CSF.

Considering that AIE is occasionally a paraneoplastic disorder, routine tumor marker measurements were performed. The results were negative, and the patient did not present with any signs of malignancy during hospitalization. After a 2-year telephone follow-up, we diagnosed the patient with gastric cancer. As previously reported, anti-DPPX antibodies are commonly associated with B-cell neoplasms. Published cases identified 1 mantle cell lymphoma, 1 B-cell lymphoma, and 1 B-cell chronic lymphocytic leukemia in 3 patients with anti-DPPX encephalitis.^[7]^ To our knowledge, this is the first reported case of anti-DPPX encephalitis in a patient with gastric cancer. Interestingly, regardless of its direct association with the generation of DPPX antibodies and gastric cancer, EBV is associated with numerous cancers, including lymphoma, nasopharyngeal carcinoma, gastric adenocarcinoma, and even breast cancer. A 2023 systematic review reported that the global prevalences of EBV in conventional gastric adenocarcinoma and lymphoepithelioma-like gastric carcinoma were 7.5% and 75.9% relatively.^[13]^ Although the specific mechanism underlying EBV infection in gastric carcinogenesis remains unclear, it is considered an oncogenic pathogen. Stomach inflammation and atrophy of gastric epithelial cells may attract B lymphocytes harboring latent EBV, thereby initiating the B-cell lytic cycle and facilitating viral transmission. Latent EBV in host cells (B lymphocytes and epithelial cells) can express small RNAs, including EBERs, EBNA1, LMP1, LMP2A, BART miRNAs, and BARF1, which may induce malignant transformation, promote cell proliferation, and resist the apoptosis of host cells by regulating the transcription of host genomes through DNA methylation, genetic mutation, or gene amplification.^[14]^ Due to the limited histopathological examination data of this patient, we could not further characterize whether gastric cancer cells were positively EB-ER hybridized, making it difficult to distinguish whether the EBV latent infection was neoplastic in this gastric carcinoma.

Owing to the long and sophisticated course of this case of encephalitis involving both viral infection and carcinoma, it is difficult to determine whether anti-DPPX encephalitis developed as a postinfectious immune process, or a paraneoplastic disorder, as the CSF findings provide only indirect evidence. Studies have shown that certain autoimmune subtypes exhibit patterns of basic CSF findings. AIE diseases with antibodies against NMDA, GABAB, and AMPA receptors as well as DPPX show frequent inflammatory CSF changes, whereas AIEs with either CASPR2, LGI1, GABAA, or glycine receptor antibodies CSF findings are mostly normal.^[15]^ Moreover, the predominance of specific IgG subclasses may be highly indicative of idiopathic origin. For example, the IgG1 subclass may be associated with robust and frequent inflammatory CSF findings, while IgG4 predominant disease subtypes rarely show an inflammatory CSF.^[15]^ While the majority of reported positive DPPX antibodies are both of the IgG4 and IgG1 subclass,^[7]^ the antibody effects were probably caused by the combined effect of IgG1 and IgG4 immunoglobulins.^[16]^ In our case, the CSF test results showed zero leukocyte count, no protein elevation, and the same oligoclonal band as the serum, which indicated a rather noninflammatory change and an impaired BBB. We inferred that DPPX might be IgG4 predominant, arising from chronic antigenic exposure late in the immune response, consistent with the long-standing stable course.

Anti-DPPX encephalitis is considered immunotherapy-responsive based on the available cases.^[9]^ First-line immunotherapy generally involves the use of corticosteroids, intravenous immunoglobulins, and plasma exchange. Our patient achieved clinical improvement after receiving intravenous methylprednisolone without seizures for more than 2 years. Unfortunately, the patient subsequently developed advanced gastric cancer, leading to a low life expectancy.

This case report has several limitations. First, we did not perform quantitative testing of EBV to evaluate reactivation in the brain; second, the DPPX subclass was not identified; and third, the lack of tumor tissue makes it difficult to determine the latency of EBV in tumor cells. Although the speculation of the exact mechanism causing encephalitis in this patient is less convincing, this case of anti-DPPX encephalitis involving latent EBV infection and gastric carcinoma provides some reference values for similar cases in clinical practice.

## 5. Conclusion

The current understanding of anti-DPPX encephalitis remains inadequate. Based on the long-term follow-up of our case, this report has the potential to expand the phenotypic spectrum and prognosis of anti-DPPX encephalitis. For patients with psychiatric symptoms, seizures, and weight loss, even those with a chronic course, it is necessary to be vigilant of the possibility of DPPX encephalitis. Further, when patients present with DPPX encephalitis, it is necessary to emphasize screening for malignant tumors, including lymphoma, nasopharyngeal carcinoma, and gastric cancer, particularly in patients with latent EBV infection. Early diagnosis of such tumors can improve the survival rates of these patients.

## Author contributions

**Data curation:** Guolu Wu, Yanhui Shi, Jialan Sun, Feifei Wu, Mei Jiang.

**Investigation:** Guolu Wu, Yanhui Shi, Feifei Wu, Mei Jiang.

**Writing – original draft:** Guolu Wu, Qiang Li.

**Methodology:** Yanhui Shi, Feifei Wu, Mei Jiang.

**Project administration:** Yanhui Shi, Jialan Sun.

**Validation:** Jialan Sun, Mei Jiang.

**Supervision:** Mei Jiang.

**Conceptualization:** Qiang Li.

**Funding acquisition:** Qiang Li.

**Writing – review & editing:** Qiang Li.
